# Subsets of regulatory T cells and their roles in allergy

**DOI:** 10.1186/1479-5876-12-125

**Published:** 2014-05-12

**Authors:** Huiyun Zhang, Hui Kong, Xiaoning Zeng, Lianyi Guo, Xiaoyun Sun, Shaoheng He

**Affiliations:** 1 Allergy and Clinical Immunology Research Centre, the First Affiliated Hospital of Liaoning Medical University, No. 2, Section 5, Renmin Street, Guta District, Jinzhou, Liaoning 121001, People’s Republic of China; 2 Central Laboratory, Suzhou Xiangcheng People’s Hospital, Suzhou 215131, China; 3 Department of Respiratory Medicine, the First Affiliated Hospital of Nanjing Medical University, Nanjing 210029, China

**Keywords:** Regulatory T cell, Allergy, IL-10, TGF-β, Mast cell

## Abstract

In recent years, it is recognized that acquired immunity is controlled by regulatory T cell (Treg). Since fundamental pathophysiological changes of allergy are mainly caused by hyperresponsiveness of immune system to allergens that acquires after birth, Tregs likely play key roles in the pathogenesis of allergy, particularly during the sensitization phase. However, accumulated information indicate that there are several distinctive subtypes of Tregs in man, and each of them seems to play different role in controlling immune system, which complicates the involvement of Tregs in allergy. The aim of the present study is to attempt to classify subtypes of Tregs and summarize their roles in allergy. Tregs should include natural Tregs (nTreg) including inducible costimulator (ICOS)(+) Tregs, inducible/adaptive Tregs (iTreg), interleukin (IL)-10-producing type 1 Tregs (Tr1 cells), CD8(+) Tregs and IL-17-producing Tregs. These cells share some common features including expression of Foxp3 (except for Tr1 cells), and secretion of inhibitory cytokine IL-10 and/or TGF-β. Furthermore, it is noticeable that Tregs likely contribute to allergic disorders such as dermatitis and airway inflammation, and play a crucial role in the treatment of allergy through their actions on suppression of effector T cells and inhibition of activation of mast cells and basophils. Modulation of functions of Tregs may provide a novel strategy to prevent and treat allergic diseases.

## Introduction

Allergic diseases are major diseases involving approximately 22% world population [1]. The diseases include allergic rhinitis, allergic asthma, allergic dermatitis, allergic conjunctitis, anaphylaxis, food or drug allergic reactions etc. It has long been accepted that allergic inflammation is the fundamental pathological changes of allergy, and type I hypersensitivity of immune system is the basic mechanism of allergic inflammation [2]. There are two phases in the basic process of IgE mediated allergic inflammation, the sensitization phase and effection phase.

It has long been recognized that lymphocytes guide (if not dictate) the sensitization of allergy by directing differentiation of uncommitted (naive) CD4 (+) T helper (Th) cells towards Th1, Th2, Th17 and Treg phenotypes. For example, the presence of IL-12 in the local milieu skews towards Th1 [expression of T box expressed in T cells (T-bet)], IL-4 towards Th2 (expression of GATA-3), transforming growth factor (TGF)-β towards Treg [expression of forkhead box P3 (Foxp3)] and IL-6 and TGF-β towards Th17 (expression of RORgammat) in murine CD4(+) T cells. It has also been demonstrated that the skewing of murine Th towards Th17 and Treg is mutually exclusive, notably the presence of IL-6 may result in a shift from a regulatory phenotype towards a Th17 [3]. It is clear that individuals with defective or suboptimal Foxp3 expression due to mutations in Foxp3 gene or in genes that promote Foxp3 expression such as STAT5b are susceptible to allergic diseases [4]. Very recently, it has been noticed that insufficient Treg and Th1 cells may be associated with the allergic inflammation that may be attributed to the Th2 immune response in patients suffering from allergic rhinitis who are sensitive to olive pollen [5].

In recent years, Tregs have been emerging as key focus in the sensitization phase of the pathogenesis of allergy. It is recognized that acquired immunity is controlled by Tregs that suppress responses of effector T cells. Tregs can be classified into nTregs [6] including inducible costimulator (ICOS)(+) Tregs [7], iTregs [4], Tr1 cells [8], CD8(+) Tregs [9] and IL-17-producing Tregs [10]. These cells share some common features including expression of Foxp3 (except for Tr1 cells), and secretion of inhibitory cytokine IL-10 and/or TGF-β (Table 1).

**Table 1:** Characteristics of subsets of regulatory T cell (Treg)

**Subset**	**Specific marker**	**Secretory products**	**Actions**	**Location**
nTreg	CD4, CD25, Foxp3	IL-10, TGF-β	Block T cell proliferation, suppression of DCs, inhibition of effector Th1, Th2, and Th17 cells; eliminate production of allergen-specific IgE, induce IgG4 secretion; suppress mast cells, basophils, and eosinophils; interact with resident tissue cells and participate tissue remodeling [12]	Thymus [9]
ICOS(+) Treg	CD4, CD25, Foxp3, ICOS	IL-10, IL-17, IFN-γ	Suppress hapten-reactive CD8(+) T cells [15]	Generated from nTregs
iTreg	CD4, Foxp3	IL-10, TGF-β	Similar to nTreg [16]	Periphery
Tr1	CD4, CD25	IL-10	Suppress effector Th cell migration and functions [4]; suppress mast cells, basophils, and eosinophils [8]	Generated from non-Treg cell precursors and home lungs and draining lymph nodes [18]
CD8(+)Treg	CD8, Foxp3, CD25 (not for tonsil origin), CD28	IL-10, TNF-α, IFN-γ, GB	Block activation of naive or effector T cells; suppress IgG/IgE antibody responses [9], IL-4 expression and the proliferation of CD4(+) T cells [19].	Generated from OT-1 CD8 cells [9]and tonsils
IL-17-producing Foxp3 (+) Treg	CD4, Foxp3,CCR6,RORGTF	IL-17	Inhibit the proliferation of CD4(+) effector T cells [10].	Differentiated from CD4(+)Foxp3(+)CCR6(-) Tregs in peripheral blood and lymphoid tissue [10]

nTreg = natural regulatory T cell; ICOS = inducible costimulator; iTreg = inducible/adaptive regulatory T cell; Tr1 cell = IL-10-producing type 1 regulatory T cell; GB = granzyme B; RORGTF = RORgammat transcription factor.

### Subsets of Tregs

At least 5 subsets of Tregs are identified so far. They are derived from naive T cells under different conditions, and play a crucial role in controlling allergic diseases.

### nTregs

The CD4(+)CD25(+)Foxp3(+) cells, which secret IL-10 and TGF-β, and represent one of the largest subsets of Treg. In mice, the cytokines associated with the Treg subset include both soluble and cell membrane-bound TGF-β and IL-10. Both contact-dependent mechanisms involving membrane-bound TGF-β to block T cell proliferation and contact-independent mechanisms involving soluble TGF-β and IL-10 have been invoked to describe the function of these Tregs [11]. These cells originate from thymus in response to self-antigens [9]. Their roles in allergen-specific immune reactions include suppression of dendritic cells that support the generation of effector T cells; inhibition of functions and migration of effector Th1, Th2, and Th17 cells; elimination of production of allergen-specific IgE and induction of IgG4 secretion; suppression of mast cells, basophils, and eosinophils; interaction with resident tissue cells and participation of tissue remodeling [12].

Very recently, CD4(+)CD25(+)CD127(lo/-) Foxp3(+) Tregs are detected in the neonatal thymus. These cells suppress the proliferative response to allogeneic stimulation of CD4(+)CD25(-) T cells dose dependently. It is also found that Treg turnover and suppressive function increases with age and paralleled the increase in global thymic Foxp3 mRNA expression in nonatopic children, whereas Treg cell maturation is significantly delayed in atopic children, implicating that the immune dysregulation in atopic children might predispose to allergic disease [13]. It has also been reported inverse correlation between expression of FoxP3 and CD127 in CD4 + CD25+ T cells, which suggests that cell surface expression of CD127 can be used as a flexible alternative to the transcription factor FoxP3 for identifying and isolating human Tregs for functional analysis [14].

ICOS(+) Tregs are distinguishable from all other Foxp3(+) Tregs by the expression of IL-10, IL-17, and interferon (IFN)-gamma. ICOS(+) Tregs derived from the expansion of nTregs rather than generation of adaptive Tregs in response to their cognate antigen. CD25(+)Foxp3(+)ICOS(+) phenotype expresses high levels of ICOS in vivo following sensitization with 2,4-dinitro-fluorobenzene, and specifically suppresses hapten-reactive CD8(+) T cells with superior suppressive activity [15].

### iTregs

In contrast to nTregs, iTregs are peripherally induced Tregs. Naive CD4+ T cells in the periphery are induced to express Foxp3 in response to foreign antigens [9] and these cells have suppressive function similar to nTregs [16]. iTregs has considerable significance in preventing asthma if generated early enough in life [4]. In addition, Th3 cells that secrete TGF-β and IL-10 belong to this subset [17].

### Tr1 cells

The CD4+ T cells that do not express Foxp3, but secret IL-10 and suppress effector functions of Th cells are known as Tr1 cells [4]. It was reported that intravenous immunoglobulin injection in OVA-sensitized and OVA-challenged mice induced production of Tr1 cells from non-Treg cell precursors. These induced Tr1 cells home specifically to the lungs and draining lymph nodes [18]. Their roles in allergen-specific immune reactions include inhibition of functions and migration of effector Th2 cells; and suppression of mast cells, basophils, and eosinophils [8].

### CD8 + Tregs

A subset of Tregs expressing CD8 is rapidly generated from OT-1 CD8 cells in the presence of IL-4 and IL-12, produce IL-10, and exhibits a unique cell-surface phenotype with coexpression of activation and naive cell-associated markers [9]. They are also observed in tonsils, but rarely detected in peripheral blood. These Foxp3(+)CD8(+) T cells can be induced in vitro in naive CD8(+) T cells by polyclonal stimulation, which express predominantly CD25(high) and CD28(high), and produce high levels of TNF-α, IFN-γ, and granzyme B. Similarly, tonsillar Foxp3(+)CD8(+) T cells also generate TNF-α and IFN-γ. However, tonsillar Foxp3(+)CD8(+) T cells do not express IL-17A, mostly CD25 negative, and express low CD127 and CD69. These Foxp3(+)CD8(+) T cells block activation of naive or effector T cells by direct T-cell-T-cell interaction that antagonizes T-cell-receptor (TCR) signals, and suppress IgG/IgE antibody responses [9], IL-4 expression and the proliferation of CD4(+) T cells [19].

### IL-17-producing Foxp3+ Tregs

It is reported that human peripheral blood and lymphoid tissue, but not thymus contain a significant number of CD4(+)Foxp3(+) T cells that express CCR6 and have the capacity to produce IL-17 upon activation. These cells coexpress Foxp3 and RORgammat transcription factors. The CCR6(+)IL-17-producing Foxp3+ Tregs strongly inhibit the proliferation of CD4(+) responder T cells. Human CCR6(+) IL-17-producing Foxp3+ Tregs are differentiated from CD4(+)Foxp3(+)CCR6(-) Tregs upon T-cell receptor stimulation in the presence of IL-1beta, IL-2, IL-21, IL-23, and human serum [10]. However, a recent report demonstrates that IL-17-producing Foxp3+ Tregs are a novel crossover immune cell population, which could be converted from Tregs to Th17 cells, and associated with a decreased suppressive function of Foxp3 CD4(+) T lymphocytes [20].

### Cell signaling of Tregs and potential mechanisms of their actions

#### Cell signaling of Tregs

**Figure 1:**
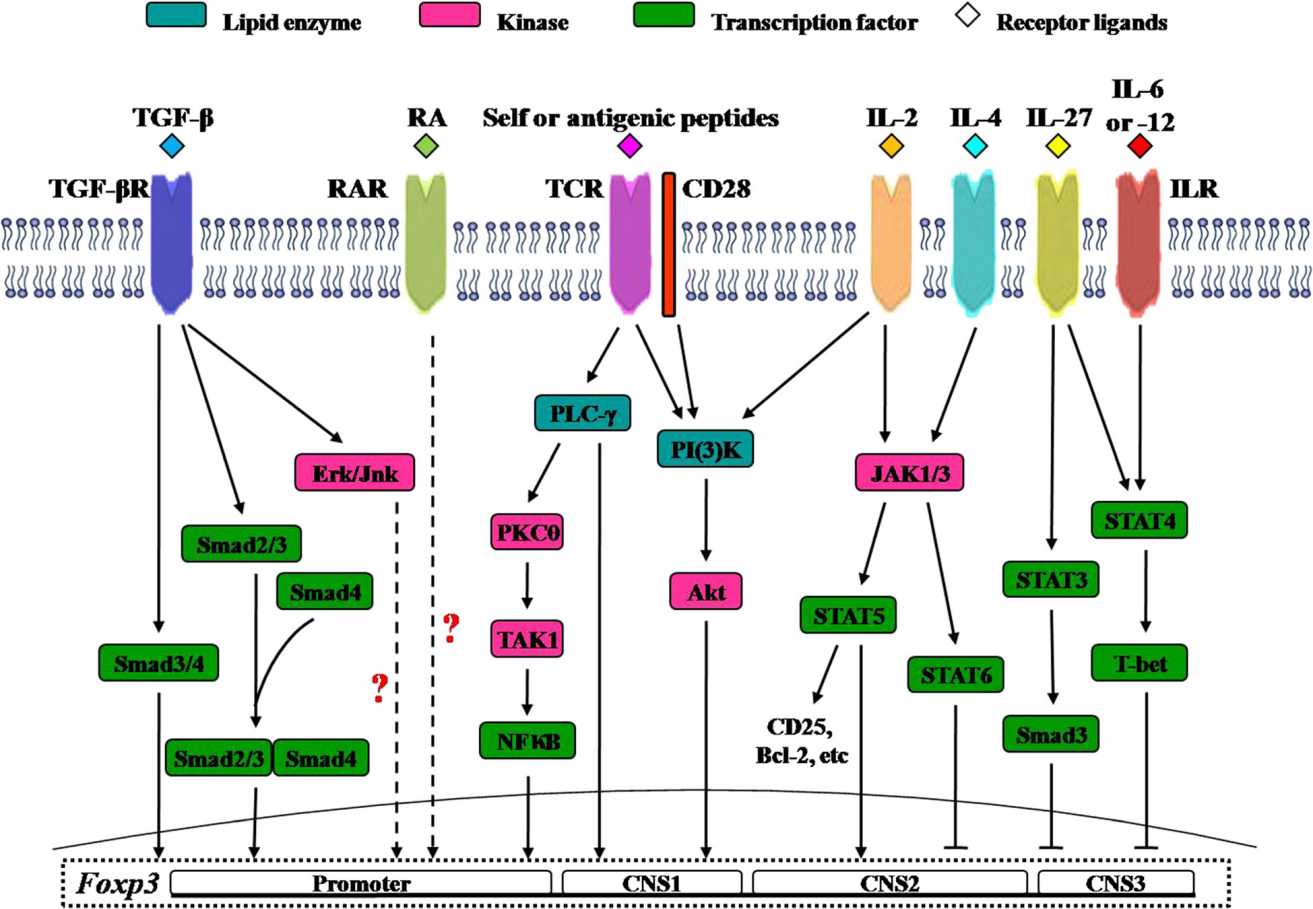
**Cell signaling pathways of Tregs.** TGF-β = transforming growth factor beta; TGF-βR = transforming growth factor beta receptor; RA = retinoic acid; RAR = retinoic acid receptor; TCR = T cell receptor; IL = interleukin; ILR = interleukin receptor; Smad = small body size and mothers against decapentaplegic; ERK = extracellular regulated protein kinase; Jnk = Jun N-terminal kinase; PLCγ = phospholipase C gamma; PKC = protein kinase C; TAK = TGF beta activated kinase; NFkB = nuclear factor kappa B; PI3K = phosphoinositide 3 kinase; AKT = protein kinase B; JAK = Janus-family tyrosine kinase; STAT = signal transducer and activator of transcription; T bet = T cell specific T-box transcription factor; Bcl-2 = B cell leukemia 2 protein; Foxp3 = forkhead box P3; CNS = non-coding sequence.

As many as 5 subsets of Tregs make Tregs as one of the most complicated T cell groups. Surely, more novel subsets of Tregs will be discovered and their biological functions will be investigated. It is very difficult to understand the reasons why so many subsets of Tregs are needed, and how these cells are generated without looking into the intracellular signaling pathways of these cells, and their migration routes. While the cellular signaling pathways and migration of Tregs remain largely uninvestigated the Figure 1 summarizes the molecular mechanisms involved in cell signaling pathways. Apart from Foxp3 mediated signaling pathways, it was reported that signal transducer and activator of transcription 4 (STAT4) is critical for IL-12 inhibited the development of TGF-β1-induced-expressing iTregs, although there is a parallel pathway involving T-bet [21]. It is found that IL-4 supersedes Treg function via the IL-4Ralpha-STAT6 axis, which decreases Foxp3 expression in Tregs and promotes the development of allergic inflammation [22]. Moreover, infectious tolerance mediated by membrane-bound TGF-β expressed on Tregs can be compromised by the competing effects of IL-4-induced signaling in naive CD4(+) Th cells [23]. Development of CD8 Tregs is directly induced via STAT4 and STAT6 signaling pathways irrespective of TCR signals [9].

### Potential mechanisms of their actions

Migration to the involved inflammatory area is one of the properties of inflammatory cells including Tregs. It has been found that Foxp3+ Tregs share major non-lymphoid tissue trafficking receptors, such as CCR4, CCR5, CCR6, CXCR3, and CXCR6 with Th17 cells, implicating that these T cells migrate to and within lymphoid tissues [24]. It has been previously summarized that the mechanisms used by Treg cells to suppress a large number of distinct target cell types can be broadly divided into those that target T cells (suppressor cytokines, IL-2 consumption, granzyme-perforin-induced apoptosis of effector lymphocytes) and those that primarily target antigen-presenting cells (decreased costimulation or decreased antigen presentation) [25]. However, anyone or more mechanisms can be used by Tregs in a defined type of inflammation.

### Actions of Tregs on other immune cells

**Figure 2:**
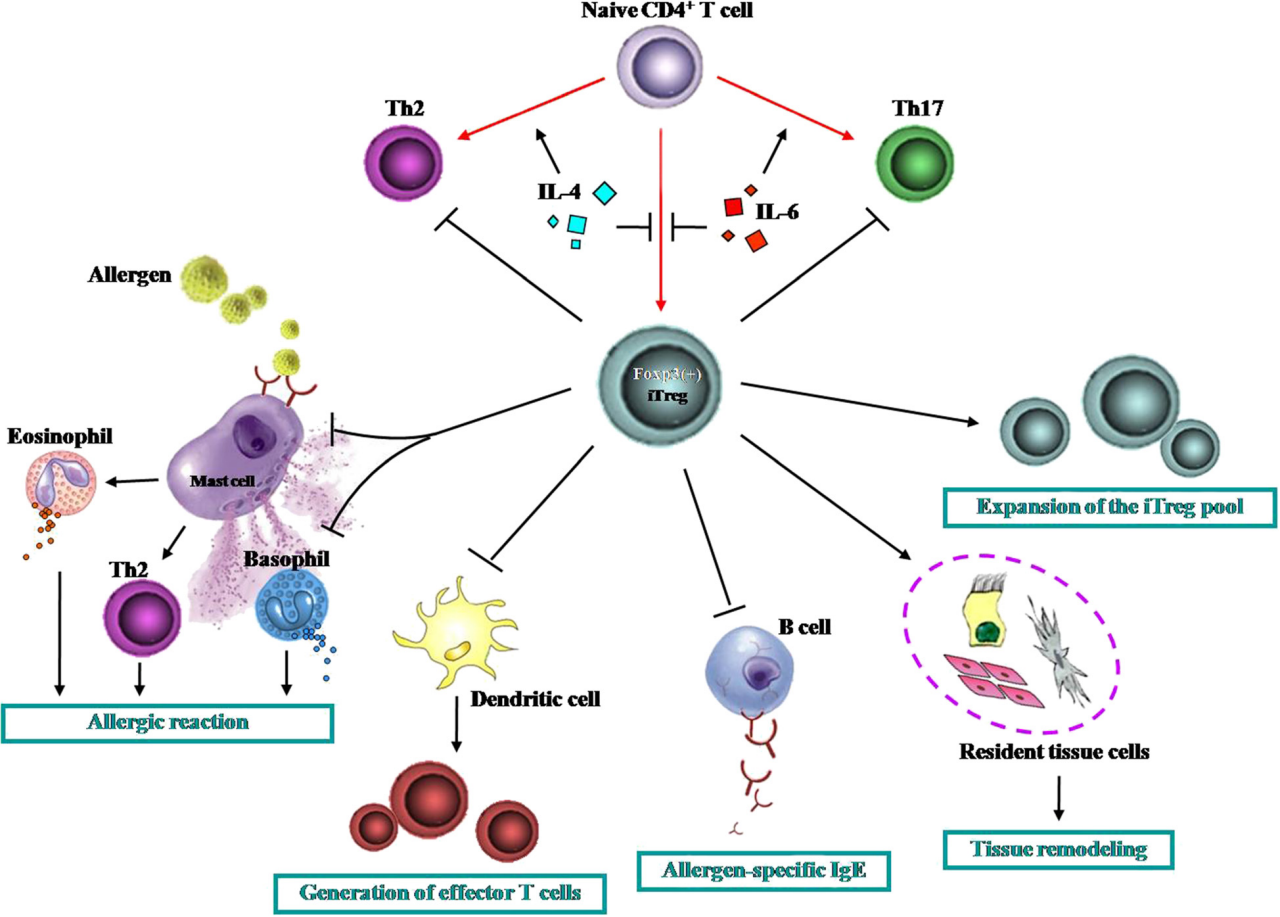
**Influence of Tregs on other cell types.** Th = helper T cells; iTreg = inducible/adaptive regulatory T cell.

The suppressive actions of Tregs on other immune cells such as effector T cells, B cells, eosinophils and mast cells may help to explain the reasons why Tregs are capable of controlling acquired immunity. In order to demonstrate the issue more clearly we summarize the actions of Tregs on other cell types in Figure 2.

### Tregs and mast cells

Mast cells are essential for immediate allergic reactions, and degranulation of this cell type is a hallmark of allergy [26]. As the classic primary effector cells, mast cells are responsible largely (if not all) for initiation of allergic pathological damage and clinical symptoms. Recently, it has been shown that constitutive Foxp3(+) Treg can control the symptomatic phase of mast cell activation and IgE-dependent anaphylaxis in mice [27], but enhance IL-6 release from mast cells. Inhibition of mast cell degranulation by Tregs appears via OX40/OX40 ligand interactions whereas inhibition of IL-6 release seems via TGF-β [28]. In vitro studies demonstrate that IL-4 and TGF-β1 had balancing effects on mast cell survival, migration, and FcepsilonRI expression, with each cytokine cancelling the effects of the other. Dysregulation of this balance may impact allergic disease and be amenable to targeted therapy [29].

### Tregs and Th cells

On activation, T cells undergo distinct developmental pathways, attaining specialized properties and effector functions. Th cells are traditionally thought to differentiate into IFN-gamma-secreting Th1, IL-4/IL-5-secreting Th2, and IL-17-secreting Th17 cells. Th1 cells are necessary to clear intracellular pathogens, Th2 cells are important for clearing extracellular antigens, and Th17 cells have been shown to have a crucial role in the induction of autoimmune tissue injury.

It has been reported that TGF-beta1 produced by Foxp3-expressing Tregs is required to inhibit Th1-cell differentiation and promote Th17-cell differentiation [30]. On the other hand, it appears that nitric oxide-induced Tregs inhibit Th17 but not Th1 cell differentiation and function [31]. Tregs can also inhibit IFN-γ synthesis without blocking Th1 cell differentiation [32]. These discriminations could be due to different experimental system being used in each study. A previous review has described that Tregs can inhibit the development of allergy via suppression of other effector Th1, Th2, Th17 cells [33]. While specific nTregs were highly effective at inhibiting the polarization of naïve CD4(+) T cells into a Th2 phenotype [34], Foxp3(+) Tregs promote Th17 cell development in vivo through regulation of IL-2 [35]. It is found that IL-6 and TGF-beta together induce the differentiation of pathogenic Th17 cells from naive T cells [36].

### Tregs and DCs

DC is the major antigen presenting cells, which play a pivotal role in the sensitization phase of allergic inflammation. It is one of the major targets of Treg-mediated suppression. Some studies have suggested that Treg-mediated suppression of DC function is mediated by the interaction of CTLA-4 on Tregs with CD80/CD86 on the DCs resulting in downregulation of CD80/CD86 expression and a decrease in costimulation. A recent study demonstrates further that the major suppressive mechanism of DC function by iTregs is secondary to the effects of IL-10 on MARCH1, an E3 ubiquitin ligase and CD83 expression [37]. Development of tolerogenic DC phenotype may be of importance for prevention of allergic contact dermatitis. Activated IL-10(+) Treg cells might induce production of the tolerogenic CD11c(+) DCs, and leads to the generation of hapten-specific CD8(+) Treg cells, which protect against contact hypersensitivity [38].

### Tregs and eosinophils

Eosinophils is one of the secondary effector cells of allergy. It is reported that Tregs are able to inhibit the activity of eosinophils [39], and a negative correlation between the number of eosinophils and the percentage of Foxp3(+) cells is found in BAL from tolerant mice [40].

### Tregs and Bregs

In recent years, regulatory B cell (Breg)s, a subset of B cells which express Foxp3 and secretion of inhibitory cytokine IL-10 and/or TGF-β have been discovered [41]. IL-10-producing B cells were the first regulatory B cells to be recognized and were termed ‘B10’ cells, which is CD19(+)CD5(+)IgM(hi)IgD(lo)CD1d(hi) type. Recently, a TGF-β-producing Breg subset Br3 has been shown to be related to immune tolerance in food allergies. Moreover, Foxp3-expressing B cells have also been identified in humans and may act as Bregs (B-Foxp3 cells). The functional image of regulatory B cells is similar to that of Tregs [42]. The Breg subtypes may be homologous to the Treg subtypes (Br1 cells expressing IL-10, Br3 cells expressing TGF-β), although the Br1 subtype seems to predominate.

Bregs act earlier, facilitating the recruitment of Tregs then disappearing once the Tregs become operational. Therefore, Bregs may play an important role in autoimmune and allergic diseases [43]. Bregs induced pulmonary infiltration of Tregs seems independent of TGF-β. Because of the proliferative and apoptotic responses of Br1 and Br3 cells in immune tolerance in non-IgE-mediated food allergy, reciprocal roles and counter-regulatory mechanisms of Br1 and Br3 responses are suspected [42]. Chronic helminth infections are associated with the expression of regulatory networks such as Tregs and Bregs necessary for downmodulating allergic immune responses to harmless antigens [44]. It is found that helminth Schistosoma mansoni infection induced IL-10-producing CD1d(high) Bregs that could prevent ovalbumin-induced allergic airway inflammation following passive transfer to ovalbumin-sensitized recipients. The transfer of Bregs can reverse established airway inflammation in ovalbumin-sensitized mice via Tregs [45].

### Contribution of Tregs to allergy

There is increasing interest in the role of both nTreg and iTreg populations in preventing hypersensitive immune responses and the underlying sensitization to allergens. As early as 2006, it was summerized that Tregs may actively prevent Th2 responses to allergens occurring in healthy non-atopic individuals and that their functions may be impaired in allergic patients [46]. It has been suggested that peripheral T-cell tolerance to environmental antigens is crucial for avoidance of allergy, and aberrant activation of Th2 cells in allergy is secondary to impaired mechanisms of peripheral T-cell tolerance that is normally mediated by antigen-specific T-cell anergy, Tregs and suppressive cytokines, IL-10 and TGF-β. Therefore, a most appealing therapy for allergic diseases would be an allergen-specific immunotherapy [47] that reduces Th2 cytokine production and promotes induction of anergy, Treg and suppressor cytokines [48]. Very recently, it is found that tonsil pDCs have the ability to generate functional CD4(+)CD25(+)CD127(-)Foxp3(+) Tregs with suppressive property from naive T cells, and that functional allergen-specific Tregs are identified both in lingual and in palatine tonsils. Since lingual tonsil is anatomically big and remains lifelong intact, it may serve as potential first-line organs of oral tolerance against allergens [49]. In order to further understand the roles of Tregs in allergy, we summarized the involvement of Tregs in different allergic diseases (Table 2).

**Table 2:** Involvement of regulatory T cell (Treg)s in different allergic diseases

**Disease**	**Location**	**Involvement of Tregs**
Allergic dermatitis	Skin, the secondary lymphoid organs	The depletion of Tregs leads to significantly exacerbated skin inflammation, as well as elevated serum IgE levels [50].
Allergic rhinitis	Tonsil, Blood	Potential first-line organs of oral tolerance against allergens [49]; allergic rhinitis patients with a good therapeutic outcome after 1 year of SIT, the induced Treg and Th1 responses persist over 3 years of SIT [52].
Allergic airway inflammation	Blood, peribronchial lymph nodes	Foxp3 expression is reduced and CD25(hi) Treg-suppressive function is deficient in asthma. Corticosteroids and allergen immunotherapy act on Tregs, in part to increase IL-10 production, while vitamin D3 and long-acting beta-agonists enhance Tr1 cell function [57]. Heligmosomoides polygyrus infection is associated with elevated numbers of Tregs in airway challenged mice [60], efficiently protects mice from asthma by induction of accumulation of highly suppressive Tregs in the lungs [61].

SIT = specific immunotherapy.

### Tregs in allergic dermatitis

Very recently, in a murine atopic dermatitis model, the numbers of nTregs are found increase in the allergen-exposed skin area and in the secondary lymphoid organs, and CD103+ effector/memory Tregs are observed to expand gradually in the lymph nodes throughout the sensitization protocol. The depletion of Tregs leads to significantly exacerbated skin inflammation, including increased recruitment of inflammatory cells and expression of Th2 cytokines, as well as elevated serum IgE levels [50]. It is also found that the ratio of Foxp3+/CD4+ cells appears skewed towards effector T cells associated with inflammation in the margin and centre of the psoriatic plaque, and that increased IL-17 is mostly related to mast cells, and only sporadically to T cells [51].

### Tregs in allergic rhinitis

A study which investigated allergen-induced Th2, Th1 and Treg immune responses in peripheral blood mononuclear cells (PBMC) and their association with symptom improvement in allergic rhinitis patients after 3 years of SIT shows that both IL-4 expression and the IL-4/IFN-gamma ratio are decreased in patients with a good therapeutic outcome after 1 year of SIT, whereas the induced Treg and Th1 responses persists over 3 years of SIT [52].

### Tregs in allergic lower airway inflammation

It was reported in 2007 that diverse populations of Tregs play an important role in regulating Th2 responses to allergens, maintaining functional tolerance. In experimental asthma models, Tregs can suppress Th2 responses to allergen, airway eosinophilia, mucous hypersecretion, and airway hyperresponsiveness (AHR) [53]. After a long-term aerosol challenge, referred to as local inhalational tolerance, ovalbumin induced allergic airway inflammation resolves with increased number of Tregs, reduced number of T and B lymphocytes in bronchoalveolar lavage fluid and hilar lymph node [54]. Depletion of Tregs during the sensitizing phase of an active immune response led to a dramatic exacerbation of allergic airway inflammation in mice, suggesting an essential role of Tregs in regulating immune responses against allergens as early as the sensitization phase via maintenance of functional tolerance [55]. Furthermore, antigen-induced airway hyperreactivity can be completely suppressed by adoptive transfer of Tregs overexpressing active TGF-β1 and IL-10 [56]. Recent work has demonstrated that Foxp3 expression is reduced and CD25(hi) Treg-suppressive function is deficient in asthma. Existing therapies including corticosteroids and allergen immunotherapy act on Tregs, in part to increase IL-10 production, while vitamin D3 and long-acting beta-agonists enhance Tr1 cell function [57]. In addition, Treg-specific ablation of the E3 ubiquitin ligase Itch in mice causes massive multiorgan lymphocyte infiltration and development of severe antigen-induced pulmonary inflammation, which unveils a mechanism of Treg acquisition of Th2-like properties that is independent of Foxp3 function and Treg cell stability [58]. All these findings indicate the pivotal role of Tregs in allergic airway inflammation. It is recognized that IL-2 is needed to maintain Foxp3+ Tregs in the periphery [4]. Therefore, a cytokine:antibody complexes of IL-2 and anti-IL-2 mAb reduce the severity of allergen-induced inflammation in the lung by expanding Tregs in vivo. Following IL-2:anti-IL-2 treatment, airway inflammation and eosinophilia is dampened, and mucus production, AHR to methacholine and parenchymal tissue inflammation are also dramatically reduced. The IL-2:anti-IL-2 treatment is dependent on Treg-derived IL-10, and suggests that endogenous Treg therapy may be a useful tool to combat asthma [59].

It has long been observed that some helminth infections are negatively associated with the prevalence of allergic disorders. Using heligmosomoides polygyrus (H. polygyrus) in a murine model of allergic airway disease and of atopic dermatitis, it is found that mice concomitantly infected with H. polygyrus diminishes eosinophil and lymphocyte recruitment into the lungs and decreased allergen-specific IgE levels. The worm infection is associated with significantly elevated numbers of Tregs in peribronchial lymph nodes in airway challenged mice. On the other hand, mast cell recruitment is significantly increased, and Tregs are basically absent in eczematous skin and remained the same in skin-draining lymph nodes in worm-infected allergic dermatitis mice, suggesting that infection with the gastrointestinal nematode leads to significant inhibition of mucosa-associated but not cutaneous allergic reactions [60]. Moreover, it has been reported recently that a bacterial pathogen Helicobacter pylori (H. pylori) infection efficiently protects mice from asthma by induction of impaired maturation of lung-infiltrating DCs and the accumulation of highly suppressive Tregs in the lungs. Since systemic Treg depletion abolishes asthma protection it suggests that the action of H. pylori is via Tregs [61]. The B subunit of E. coli heat-labile enterotoxin (EtxB) treatment diminishes eosinophilia in bronchoalveolar lavage, reduces OVA-specific IgE and IL-4 production locally and systemically, and reduces AHR in a mouse model of asthma. EtxB induces a dose-dependent increase in OVA-specific CD4(+)Foxp3(+) T cells in the lung and systemically, suggesting that the action of. EtxB on the mice is through iTregs [62].

### Roles of Tregs in treatment of allergy

It has been recognized that Tregs are involved in the treatment of allergic diseases, which helps to explain some therapeutic mechanisms that puzzle us for decades. The potential roles of Tregs in anti-allergic therapies are summarized in Table 3.

**Table 3:** Therapeutic actions of regulatory T cell (Treg)s in allergic diseases

**Therapy**	**Action of Tregs**
SIT	Suppression of T cell responses to the T-cell epitopes of major allergens. Autocrine action of IL-10 and/or TGF-β, which are produced by antigen-specific Tregs. They may suppress IgE production and induce IgG4 and IgA production against allergens [65]. Histamine released from mast cells and basophils may efficiently contribute to immunoregulation, and affect Tregs [6]. Der p immunotherapy causes increased number of Treg cells, and elevated IL-10 production. IL-10(+) Tregs may respond to Der p-2 and down-regulate NF-κB/p65 expression in PBMC to maintain immune tolerance during SIT [66].
SLIT	Allergen extracts administered via the sublingual route are long retained at mucosal level, where the allergen molecules are captured by DCs, following their migration in the draining lymph nodes, presented to T cells to generate iTregs [64]. SLIT causes the absence of effectors cells, such as mast cells, basophils and eosinophils in the oral mucosa of allergic subjects. Skewing of allergic-specific effector T cells to a Tr1 phenotype appears to be a critical event in successful allergen-specific immunotherapy and glucocorticoids and beta2-agonists treatment [8].
Bacteria therapy	Lactobacilli prime of DCs to drive the development of Tregs. These Tregs produce increased IL-10 inhibiting the proliferation of bystander T cells [4].
Treg therapy	Transfer of OVA peptide-specific CD4 + CD25+ Tregs to OVA-sensitized mice reduces AHR, recruitment of eosinophils, and Th2 cytokine expression in the lung after allergen challenge [70].

SIT = specific immunotherapy; Der p = Dermatophagoides pteronyssinus; PBMC = peripheral blood mononuclear cells; SLIT = sublingual immunotherapy; AHR = airway hyperresponsiveness; OVA = ovalbumin.

### In specific immunotherapy

Antigen specific respiratory tolerance is mediated by lung DCs producing IL-10, which induce the development of Tregs. The development of respiratory tolerance also depends on co-stimulation (CD86, and the ICOS-ICOSL pathway). Although exposure of the respiratory mucosa to some pathogenic agents (especially virus, and endotoxin) is associated with asthma exacerbations, microbial exposure may also promote mucosal tolerance and protection against the development of allergic diseases. Mucosal-based immunotherapy has been already used as an alternative form of allergen delivery in immunotherapy [63]. Allergen-specific immunotherapy is the only treatment that leads to reverse established allergic disease and lifelong tolerance to previously disease-causing allergens by restoring normal immunity against allergens. Tregs are involved in preventing sensitization to allergens by suppression of T cell responses to the T-cell epitopes of major allergens. It is initiated by the autocrine action of IL-10 and/or TGF-β, which are produced by antigen-specific Tregs. They may suppress IgE production and induce IgG4 and IgA production against allergens. In addition, histamine released from mast cells and basophils may efficiently contribute to immunoregulation during specific immunotherapy, and affect Treg cells and the production of their cytokines via histamine receptor 2 [6].

Sublingual immunotherapy (SLIT) is an effective and safe treatment for respiratory allergy. Studies of pharmacokinetics shows that allergen extracts administered via the sublingual route are not directly absorbed by the oral mucosa but are long retained at mucosal level, where the allergen molecules are captured by DCs, following their migration in the draining lymph nodes, presented to T cells to generate iTregs. This is at least for the long-term effects, and for the short-term efficacy, observed with ultrarush or coseasonal treatment, a down-regulation of mast cells resulting in hyporeactivity at the peak of the pollen season may be suggested [64]. Indeed, SLIT causes the absence of effectors cells, such as mast cells, basophils and eosinophils in the oral mucosa of allergic subjects. Skewing of allergic-specific effector T cells to a Tr1 phenotype appears to be a critical event in successful allergen-specific immunotherapy and glucocorticoids and beta2-agonists treatment. Tr1 suppresses Th2 cells and effector cells of allergic inflammation, such as eosinophils, mast cells, basophils, through producing IL-10 [8], and TGF-β [65]. The increased levels of IL-10 and TGF-β that are produced by Tregs potently suppress IgE production, while simultaneously increasing production of non-inflammatory isotypes IgG4 and IgA, respectively [65]. NF-κB acts as a mater switch for allergic inflammation. Dermatophagoides pteronyssinus (Der p) immunotherapy causes increased numbers of Foxp3+ CD4+ Tregs, and elevated IL-10 production with decreased IRAK-1 and NF-κB/p65 nuclear translocation. IL-10(+) Tregs may respond to Der p-2 and down-regulate NF-κB/p65 expression in peripheral blood mononuclear cells to maintain immune tolerance during immunotherapy [66].

### In other therapies

Parasite infection does not prevent allergen sensitisation, but restricts the Th2 effector phase responsible for inflammation. Suppression of allergic inflammation can be transferred by Tregs from an infected, allergen-naïve animal to an uninfected, sensitized recipient [67]. This may due to at least in part that infections increase the activity of Tregs, which suppress effector mechanisms of both Th1 and Th2 types [68].

Using green fluorescent protein (GFP)-Foxp3 knock-in reporter mice, it is found that intravenous immunoglobulin (IVIG) markedly improves OVA-induced airway hyperresponsiveness characterized by 4- to 6-fold enhancement in Tregs in pulmonary and associated lymphoid tissues. Induction of Tregs is mediated by tolerogenic DCs generated after IVIG exposure. IVIG-primed DCs express altered Notch ligands, including increased Delta-4 and reduced Jagged-1 levels, reflecting decreased Th2 polarization. Furthermore, IVIG-primed DCs can stimulate Treg differentiation from uncommitted Foxp3(-)CD4(+) T cells ex vivo [18]. It is now well recognized that microorganisms can induce the generation of suppressive cytokines TGF-beta and IL-10 [4]. Two different species of lactobacilli, Lactobacillus reuteri and Lactobacillus casei are able to prime of monocyte-derived immunomodulation DCs to drive the development of Tregs by binding the C-type lectin DC-specific intercellular adhesion molecule 3-grabbing non-integrin (DC-SIGN). These Tregs produce increased level of IL-10 and are capable of inhibiting the proliferation of bystander T cells in an IL-10-dependent fashion. The probiotic bacteria might be used in the treatment of a number of inflammatory diseases, including atopic dermatitis and Crohn’s disease [69].

Transfer of OVA peptide-specific CD4 + CD25+ Tregs to OVA-sensitized mice reduces AHR, recruitment of eosinophils, and Th2 cytokine expression in the lung after allergen challenge. This suppression is dependent on IL-10 because increased lung expression of IL-10 is detected after transfer of Tregs, and regulation is reversed by anti-IL-10 receptor antibody. Tregs can suppress the Th2 cell-driven response to allergen in vivo by an IL-10-dependent mechanism but that IL-10 production by the Tregs themselves is not required for such suppression [70].

### Tregs in early life

The concept of iTregs has considerable significance in preventing asthma if generated early enough in life. This is because cytokines such as IL-4 and IL-6 inhibit Foxp3 induction in naive CD4+ T cells and therefore de novo generation of Tregs can be expected to be less efficient when it is concomitant with effector cell development in response to an allergen. However, if iTregs can be induced, the process of infectious tolerance would facilitate expansion of the iTreg pool. There may be an opportunity in early life in the context of a relatively immature immune system that is permissive for the generation of iTregs specific to a spectrum of allergens that would regulate asthma for lifelong [4]. A recent study shows that the timothy- and birch-allergic mothers respond with increased proliferation and/or IL-4 production towards timothy and birch extract. However, cord blood mononuclear cell proliferation, phenotype of T cells, Foxp3(+) Tregs and B cells in response to the allergens are not affected by the allergic status of the mother [71]. Fetal exposures have been investigated as risk factors of early life allergic disease, and it is showed that nTreg levels during pregnancy in venous blood samples vary in association with both dog and cat exposure and atopic status [72].

### Summary

Tregs have been emerging as key focus in the sensitization phase of the pathogenesis of allergy. Through acting upon other cell types, Tregs seem to control acquired immunity in the body. Based upon the information available, we propose that Tregs should be classified into 5 subgroups: nTregs including ICOS)(+) Tregs, iTregs, Tr1 cells, CD8(+) Tregs, and IL-17-producing Tregs. These cells share some common features including expression of Foxp3, and secretion of inhibitory cytokine IL-10 and/or TGF-β. However, signaling pathways of Tregs remain largely uninvestigated.

In recent years, there is increasing interest in the role of both nTreg and iTreg populations in preventing hypersensitive immune responses and the underlying sensitization to allergens. Since eliminated actions of Tregs may increase the chance for sensitive individuals to suffer from allergy, we summarized the involvement of Tregs in different allergic diseases. It has been suggested that peripheral T-cell tolerance to environmental antigens is crucial for avoidance of allergy. Therefore a most appealing therapy for allergic diseases would be SIT that reduces Th2 cytokine production and promotes induction of T cell anergy, Tregs and suppressor cytokines. Tregs are also involved in other immune therapies such as parasite or bacterial infections to treat allergy.

In conclusion, at least 5 subsets of Tregs are derived from naive T cells under different conditions, but exact role of each subtype of them in controlling allergic diseases remains obscure. It is widely accepted that Tregs play a pivotal role in the development of allergy, particularly in the sensitization phase. Therefore targeting Tregs can be a useful therapy for prevention and treatment of allergy.

## Competing interests

The authors declare that there is no competing interests regarding the publication of this article.

## Authors’ contribution

HY Z created the tables and drafted the primary manuscript. H K and XN Z drew the figures. LY G and XY S collected some data. SH H designed and corrected the manuscript. All authors have read and approved the final manuscript.
